# A Twisted Tale: A Case of Intestinal Volvulus in an 85‐Year‐Old Female

**DOI:** 10.1155/crip/6843513

**Published:** 2025-12-22

**Authors:** Henry Elsenpeter, Paxten Wahlund, Selly Strauch, Susan Roe

**Affiliations:** ^1^ Department of Pathology, School of Medicine & Health Sciences, University of North Dakota, Grand Forks, North Dakota, USA, und.edu

**Keywords:** case report, forensics, small bowel obstruction, volvulus

## Abstract

Medicolegal death investigation (MDI) includes sudden and unexpected deaths, with the most common being cardiovascular deaths, cerebral, pulmonary, and cancer causes. Less commonly, gastrointestinal conditions can occur, and most conditions are symptomatic. Occasionally, pain is masked or absent. Of the many intestinal conditions that may cause death, intestinal volvulus occurs in all ages and is part of the larger intestinal obstruction disease states. The individual in our report was an active, articulate 85‐year‐old female in excellent health; her medical history was positive only for hypothyroidism, treated for over 30 years with Synthroid. She died following vague abdominal complaints and nausea following a meal. At autopsy, a volvulus of the ileum and adjacent mesentery was present. The ileum was necrotic, and 700 cc of bloody, dark peritoneal fluid was present. Major arteries, including celiac, superior, and inferior mesenteric arteries, were patent. A volvulus occurs when the bowel twists upon its associated mesentery, causing obstruction within the intestine, vascular compromise of the supporting vessels in the mesentery, and ultimate distal ischemia of the affected bowel. Correct assessment in this case provided answers for the family and allowed correct death certificate classification.

## 1. Introduction

Volvulus occurs when the bowel twists upon its mesenteric point of attachment, resulting in both luminal and vascular compromise. The flow of intestinal contents is then obstructed, and the subsequent clamping of blood supply to and from the area results in ischemia. The obstruction increases the risk of perforation of the bowel, while hypoxia raises concerns for necrosis [1]. The pathophysiology of mechanical bowel obstruction is dilation of the proximal area to the obstruction. The buildup of contents results in distention of the bowel and eventual edema, which itself results in loss of absorptive capacity. This then leads to electrolyte abnormalities, which are further exacerbated by the associated vomiting from the small bowel obstruction (SBO). Strangulation happens when intraluminal pressure is greater than perfusion pressure. Once perfusion cannot be achieved, ischemia and eventual necrosis occur. Mortality is greatly increased with necrosis [2]. Closed‐loop volvulus differs from simple mechanical obstruction by containing relatively little gas and involving venous stasis. Venous stasis results in the extravasation of blood and plasma into the loop and surrounding mesentery, which leads to a much more rapid pace of intestinal distension. Strangulation is almost always secondary to a closed‐loop obstruction accompanied by adhesions or herniation [3].

The symptoms are typically constant when ischemia occurs, with severe abdominal pain. Prior to ischemia, there may only be crampy intermittent abdominal pain [2]. Patients may also present with abdominal distension, vomiting, bloody stools, constipation, fever, tachypnea, and/or tachycardia [1]. While colonic volvulus is the third most common cause of acute colonic obstruction in the world, small bowel volvulus (SBV) itself is rare, representing only 1% of the etiologies of SBO [4, 5]. Even more rare is the incidence of SBV in adults. In pediatric patients, one in 500 will have a malrotation, with 80% of cases resulting in SBV. The annual incidence of SBV among adults in Western countries is 1.7–5.7 per 100,000. Interestingly, the annual incidence of SBV is 24–60 per 100,000 adults in Africa, the Middle East, and Asia. Serotonin‐rich diets, fasting, and fiber‐rich diets are believed to play a role in explaining this disparity. The most common source of the volvulus in the adult population is thought to be adhesive disease, which one retrospective analysis found in 32.44% of cases. The same study reported a mortality rate for general bowel obstruction of 5.61% with an overall mortality rate for patients with SBV of 7.92% [6].

## 2. Case Report

The patient was an 85‐year‐old female with a past medical history of hypothyroidism for which she took Synthroid for 30 years. There was no personal history of hypertension, coronary artery disease, diabetes, or lipid disorders. There was no prior history of abdominal surgeries, and the family history was only positive for hypertension. She presented to her local hospital after waking up with lower abdominal pain, vomiting, and nausea. The pain was unresponsive to ibuprofen and eventually began to radiate from both lower abdominal quadrants to her lower back.

Computed tomography (CT) of the abdomen and pelvis found a SBO without free air, small amounts of ascites, and a hiatal hernia. There were multiple, dilated small bowel loops. There was a transition point in the lower right quadrant. Her distal esophagus had a fluid‐filled hernia sac. She was transferred to a larger hospital for a surgical consultation due to concern for a SBO. Her labs were remarkable for WBC of 14,200 cells/*μ*L, hemoglobin of 17.5 g/dL, platelets of 267 K/uL, glucose of 227 mg/dL, serum sodium of 133 mEq/L, and no liver function test abnormalities. Surgery consultation noted no peritoneal signs and only mild tenderness. She was maintained on bowel rest, Zofran, Dilaudid, and intravenous fluids. The surgeon recommended a CT scan with oral contrast the following day and supportive care. She vomited twice early the following morning. At 9 a.m., she was found sitting on the ground after falling backward and striking her head. A CT of the head found no acute intracranial abnormalities. At 3 p.m., she was found kneeling on the ground and vomiting feculent material. A code blue was called after central pulses were lost. After 13 min of cardiopulmonary resuscitation, a return of spontaneous circulation was achieved. She was transferred to the intensive care unit and deteriorated neurologically. She was switched to comfort measures and passed away that night.

At autopsy, the patient appeared normally developed and normally nourished. The abdomen was mildly protuberant and discolored green.

**Figure 1:**
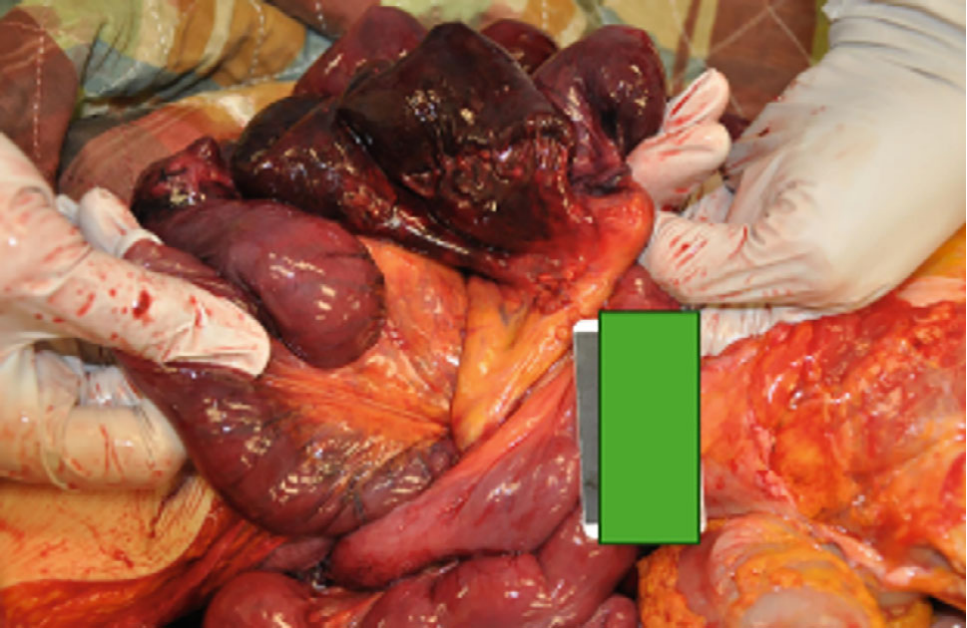
Necrotic ileum and area of near bowel perforation, with twisting of the mesentery.

**Figure 2:**
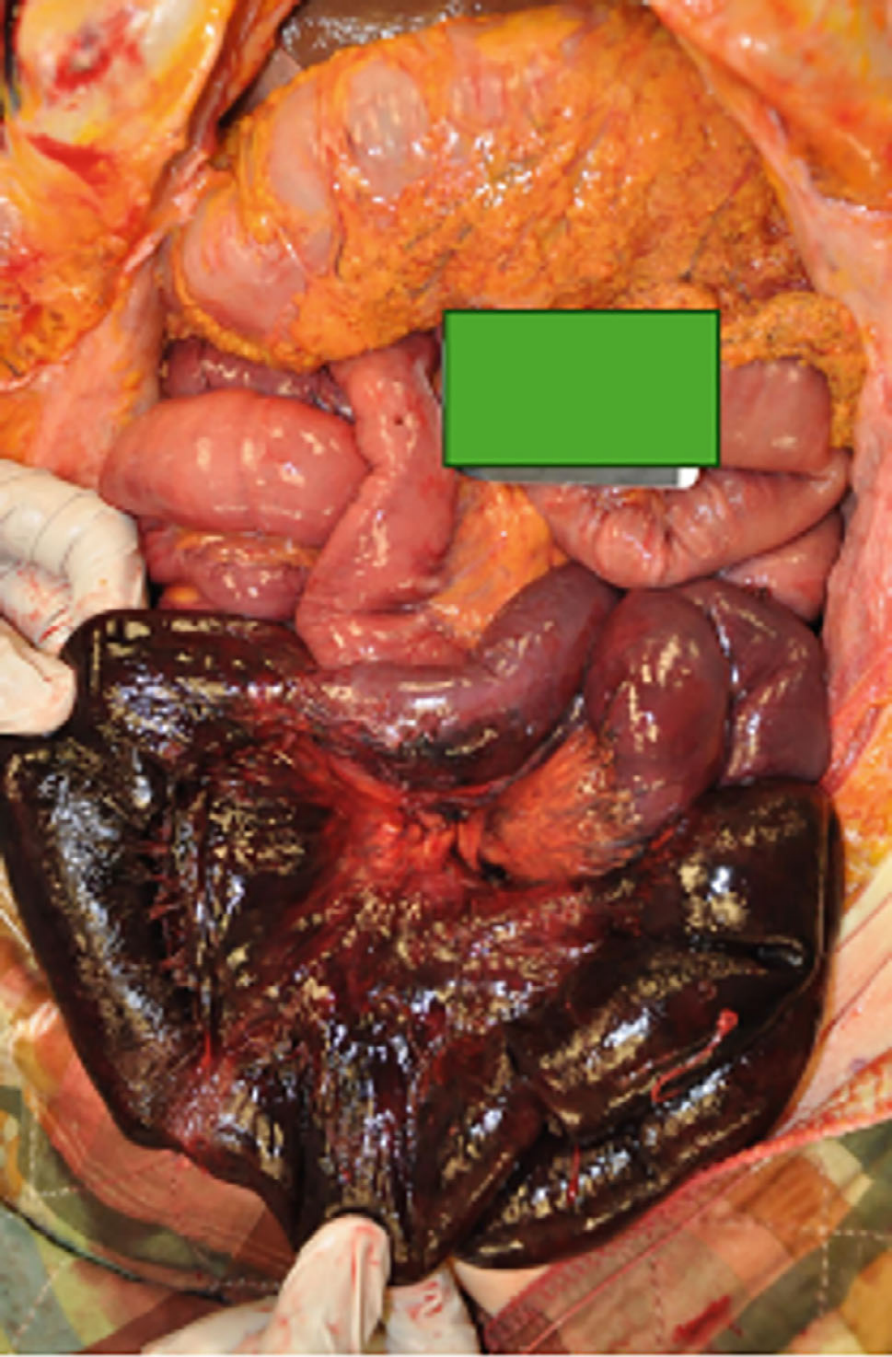
Gross appearance of necrotic ileum with twisting of the mesentery.

**Figure 3:**
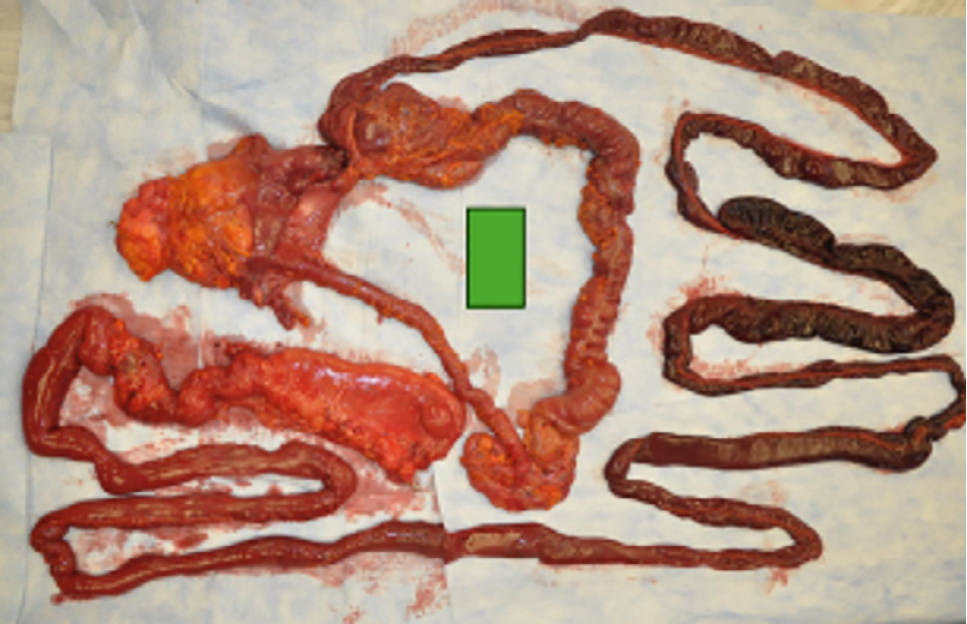
Length of entire dissected gastrointestinal tract.

There was a volvulus present in the mid‐ileum. The ileum proximal to the constriction was darker in appearance and was black in regions. The wall was thin with perforation of the bowel (see Figure 1). The ileum and its adjacent mesentery were twisted, resulting in both ischemia and necrosis of the ileum (see Figures 2 and 3). There were no masses. Upon internal exam, the superior and inferior mesenteric arteries and the celiac artery were patent. In the distal large bowel, there was formed stool past the splenic flexure. Bloody liquid was present in the ileum. There were 700 cc of bloody, dark peritoneal fluid. The cecum was hyperemic, with focal hemorrhage around the appendix. There was no ischemia or necrosis noted in this area. Microscopic evaluation of the involved ileum demonstrated transmural necrosis.

The cause of death was listed as volvulus with necrotic ileum. The manner of death was classified as natural for public health reasons.

## 3. Discussion

SBV is a rare condition in adults. It is characterized by a segment of small intestine twisting upon its point of mesenteric attachment, thereby compromising luminal flow of intestinal contents, flow of blood to the bowel, and venous outflow.

If volvulus is not recognized, it may progress to necrosis of the bowel and death of the patient over the course of several days. A study of 20,680 patients with SBV documented that the mean time from admission to death was 8.11 ± 11.6 days (range: 0–209 days). Our case diverged from this trend with a sudden and unexpected course prompting the medicolegal death investigation. However, demographically, this case of an 85‐year‐old female was consistent with other findings reported in the literature. The majority of SBV patients are female, and the mean age is 66.0 ± 19.4 years. The data is unclear if this is specific to SBV or if it is simply a reflection of the demographics of bowel obstruction at large, which also tends to be more common in elderly females [6]. Another factor to consider is anatomical predisposition for bowel twisting. Secondary SBV due to postoperative adhesions, congenital abnormalities, or neoplasm is much more common than primary SBV [5]. However, when it comes to cases of SBO in patients without known pathology or previous operation, a scoping review by the *World Journal of Emergency Surgery* identified adhesions as the cause of obstruction in approximately half of reported cases [7].

The nonspecific symptoms of SBV and its rarity may result in a delayed diagnosis. Such a delay increases both mortality and morbidity [8].

This case highlights the importance of medicolegal death investigation. Without investigation, the patient′s death would likely have been incorrectly ascribed to another cause. Consideration was given to this patient′s history of eating at a community event at her church prior to her presentation with abdominal complaints and nausea. There were public health concerns of exposure to a food‐borne illness. Timely investigation of a food‐borne illness is necessary to isolate the source of contamination and stop the spread of the contaminant. Medicolegal death investigation allows accurate diagnosis and etiologies of unclear or unexpected deaths that affect not only vital statistics but also public health.

## 4. Conclusion

This is a report of a sudden and unexpected case of SBV in an 85‐year‐old female. The rapid presentation and recent food consumption at a community event make this an unusual presentation. SBV is rare, and its onset is often characterized by nonspecific findings, contributing to difficulties with diagnosis. Awareness of the signs and symptoms of volvulus presentation may lead to increased awareness, leading to more timely management.

## Conflicts of Interest

The authors declare no conflicts of interest.

## Funding

No funding was received for this manuscript.

## Data Availability

The data that support the findings of this study are available upon request from the corresponding author. The data are not publicly available due to privacy or ethical restrictions.

## References

[1] Le C. K. , Nahirniak P. , Anand S. , and Wantzy C. , Volvulus Continuing Education Activity, 2022, StatPearls Publishing, Available from: https://www.ncbi.nlm.nih.gov/books/NBK441836/?report=printable.

[2] Butler K. L. and Harisinghani M. G. , Acute Care Surgery: Imaging Essentials for Rapid Diagnosis, 2015, 1st edition, McGraw-Hill Education.

[3] Mbengue A. , Ndiaye A. , Soko T. O. , Sahnoun M. , Fall A. , Diouf C. T. , Régent D. , and Diakhaté I. C. , Closed Loop Obstruction: Pictorial Essay, Diagnostic and Interventional Imaging. (2015) 96, no. 2, 213–220, 10.1016/j.diii.2013.10.011, 2-s2.0-84952322833, 24290342. 24290342

[4] Perrot L. , Fohlen A. , Alves A. , and Lubrano J. , Management of the Colonic Volvulus in 2016, Journal of Visceral Surgery. (2016) 153, no. 3, 183–192, 10.1016/j.jviscsurg.2016.03.006, 2-s2.0-85020292819, 27132752. 27132752

[5] Bouassida M. , Beji H. , Chtourou M. F. , Ben Othmane N. , Hamzaoui L. , and Touinsi H. , Primary Small Bowel Volvulus: A Case Report and Literature Review, Annals of Medicine and Surgery. (2022) 80, 10.1016/j.amsu.2022.104250, 36045801. PMC9422278 36045801

[6] Coe T. M. , Chang D. C. , and Sicklick J. K. , Small Bowel Volvulus in the Adult Populace of the United States: Results From a Population-Based Study, American Journal of Surgery. (2015) 210, no. 2, 201–210.e2, 10.1016/j.amjsurg.2014.12.048, 2-s2.0-84931563233, 26002189. 26002189 PMC4475430

[7] Amara Y. , Leppaniemi A. , Catena F. , Ansaloni L. , Sugrue M. , Fraga G. P. , Coccolini F. , Biffl W. L. , Peitzman A. B. , Kluger Y. , Sartelli M. , Moore E. E. , di Saverio S. , Darwish E. , Endo C. , van Goor H. , and ten Broek R. P. , Diagnosis and Management of Small Bowel Obstruction in Virgin Abdomen: A WSES Position Paper, World Journal of Emergency Surgery : WJES. (2021) 16, no. 1, 10.1186/s13017-021-00379-8, 34217331. PMC8254282 34217331

[8] Li R. , Quintana M. T. , Lee J. , Sarani B. , and Kartiko S. , Timing to Surgery in Elderly Patients With Small Bowel Obstruction: An Insight on Frailty, Journal of Trauma and Acute Care Surgery. (2024) 97, no. 4, 623–630, 10.1097/TA.0000000000004410, 38787701. 38787701

